# Fine mapping of the celiac disease-associated *LPP* locus reveals a potential functional variant

**DOI:** 10.1093/hmg/ddt619

**Published:** 2013-12-11

**Authors:** Rodrigo Almeida, Isis Ricaño-Ponce, Vinod Kumar, Patrick Deelen, Agata Szperl, Gosia Trynka, Javier Gutierrez-Achury, Alexandros Kanterakis, Harm-Jan Westra, Lude Franke, Morris A. Swertz, Mathieu Platteel, Jose Ramon Bilbao, Donatella Barisani, Luigi Greco, Luisa Mearin, Victorien M. Wolters, Chris Mulder, Maria Cristina Mazzilli, Ajit Sood, Bozena Cukrowska, Concepción Núñez, Riccardo Pratesi, Sebo Withoff, Cisca Wijmenga

**Affiliations:** 1 Department of Genetics, University of Groningen, University Medical Center Groningen, PO Box 30001, Groningen 9700 RB, The Netherlands; 2 Graduate Program in Health Sciences, University of Brasilia School of Health Sciences, Brasilia, Brazil; 3 Immunogenetics Research Laboratory, Hospital Universitario de Cruces, Barakaldo, Bizkaia 48903, Spain; 4 Department of Experimental Medicine, Faculty of Medicine, University of Milano-Bicocca, Monza, Italy; 5 European Laboratory for Food Induced Disease, University of Naples Federico II, Naples, Italy; 6 Department of Pediatric Gastroenterology, Leiden University Medical Centre, Leiden, The Netherlands; 7 Department of Pediatric Gastroenterology, University Medical Centre Utrecht, Utrecht, The Netherlands; 8 Department of Gastroenterology, VU Medical Center, Amsterdam, The Netherlands; 9 Department of Molecular Medicine, Sapienza University of Rome, Rome, Italy; 10 Dayanand Medical College and Hospital, Ludhiana, Punjab, India; 11 Department of Pathology, Children's Memorial Health Institute, Warsaw, Poland; 12 Depatment of Immunology, H. Clínico S. Carlos, Instituto de Investigación Sanitaria San Carlos (IdISSC), Madrid, Spain

## Abstract

Using the Immunochip for genotyping, we identified 39 non-human leukocyte antigen (non-HLA) loci associated to celiac disease (CeD), an immune-mediated disease with a worldwide frequency of ∼1%. The most significant non-HLA signal mapped to the intronic region of 70 kb in the *LPP* gene. Our aim was to fine map and identify possible functional variants in the *LPP* locus. We performed a meta-analysis in a cohort of 25 169 individuals from six different populations previously genotyped using Immunochip. Imputation using data from the Genome of the Netherlands and 1000 Genomes projects, followed by meta-analysis, confirmed the strong association signal on the *LPP* locus (rs2030519, *P* = 1.79 × 10^−49^), without any novel associations. The conditional analysis on this top SNP-indicated association to a single common haplotype. By performing haplotype analyses in each population separately, as well as in a combined group of the four populations that reach the significant threshold after correction (*P* < 0.008), we narrowed down the CeD-associated region from 70 to 2.8 kb (*P* = 1.35 × 10^−44^). By intersecting regulatory data from the ENCODE project, we found a functional SNP, rs4686484 (*P* = 3.12 × 10^−49^), that maps to several B-cell enhancer elements and a highly conserved region. This SNP was also predicted to change the binding motif of the transcription factors IRF4, IRF11, Nkx2.7 and Nkx2.9, suggesting its role in transcriptional regulation. We later found significantly low levels of *LPP* mRNA in CeD biopsies compared with controls, thus our results suggest that rs4686484 is the functional variant in this locus, while *LPP* expression is decreased in CeD.

## INTRODUCTION

In recent years, genome-wide association studies (GWAS) have identified thousands of new susceptibility loci for common diseases, including dozens for celiac disease (CeD) (1,2). CeD is an immune-mediated disease triggered by gluten in the diet of genetically susceptible individuals, and it is strongly correlated with the presence of specific HLA-DQ isotypes, which are necessary but not sufficient to lead to the disease (3). In a previous GWAS, we identified 26 non-human leukocyte antigen (non-HLA) loci with small effect size that explain part of the heritability of CeD (4). Recently, we used the Immunochip (5) and discovered 13 additional susceptibility loci, as well as fine mapping >50% of all 40 loci (6). In total, we reported 39 non-HLA susceptibility loci, which together with the HLA locus, explain 50% of CeD heritability (6). The strongest non-HLA association signal maps to a 70 kb linkage disequilibrium (LD) block in intron 2 of the *LPP* gene. Conditional analysis of this highly associated variant revealed no other signals of association, indicating a single-associated haplotype in this locus.

The LPP gene has also been reported to be associated with vitiligo (7), an autoimmune disease, in which the top SNP is located in the same haplotype as the CeD top SNP (*r*^2^ = 0.8 and *D*′ = 0.9). The LPP gene is involved in cell motility and cell–cell adhesion, which is crucial to maintaining the barrier integrity of epithelial monolayers, especially in the small intestine. It might therefore play a role in the pathogenesis of CeD (8,9).

Our aim was to fine map the CeD-associated 70 kb intronic region at the *LPP* locus and to identify possible functional variants. We used data from six different CeD populations (UK, Dutch, Polish, Spanish, Italian and Indian) genotyped on the Immunochip array. Further, a haplotype analysis was performed, followed by haplotype association testing in each population separately. Finally, we intersected the fine-mapped region with functional annotation data from the ENCODE project (10). This allowed us to refine the 70 kb region of association to a 25-fold smaller region of 2.8 kb enriched with regulatory elements, where we identified one SNP predicted to change transcription-factor-binding motif.

## RESULTS

### Meta-analysis identified a single variant at *LPP* locus

**Figure 1.**
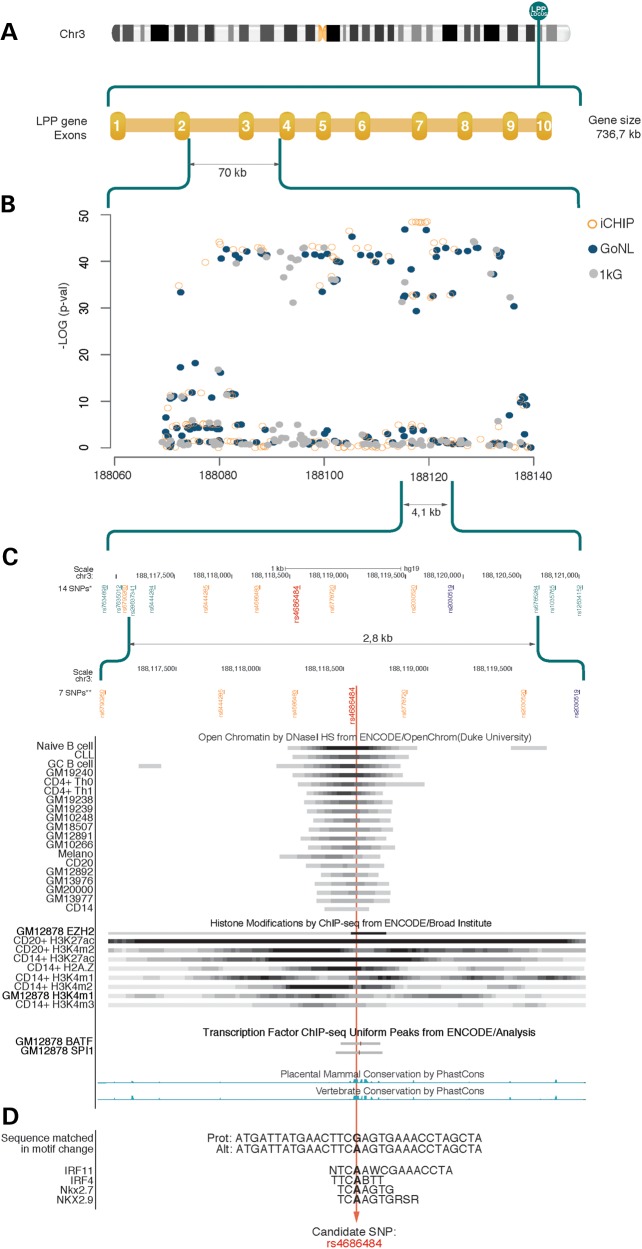
Fine mapping of the *LPP* region. (**A**) Localization of the *LPP* gene on chromosome 3 and the LD region. (**B**) Meta-analysis of imputation and Immunochip (iCHIP) results. Open circles are SNPs genotyped by the iCHIP, black dots represent SNPs imputed by GoNL and gray dots represent SNPs imputed by the 1000 Genomes Project (1kG). (**C**) SNPs in the fine-mapping region overlapping to ENCODE regulatory regions, including DNase I hypersensitive sites, histone modifications determined by ChIP-seq and transcription factor-binding site ChIP signals. Signal peaks are depicted as darker regions. Evolutionary conserved sites are also shown. *14 SNPs that construct the core-haplotype. **7 SNPs in orange differentiate the risk from the non-risk haplotype. The functional candidate SNP rs4686484 is depicted in red. The top meta-analysis SNP is in blue. (**D**) Sequencing of the functional candidate SNP predicted to change the binding site of the shown transcription factors. Prot, protective; Ref, reference.

In order to fine map the 70 kb *LPP* LD region, we performed a meta-analysis on a large cohort of 25 169 individuals, who had been genotyped by Immunochip (Table 1, Supplementary Material, Table S1). The SNP with the highest association with CeD was located within intron 2 of the *LPP* locus (rs2030519; *P* = 1.76 × 10^−49^; OR = 0.75, 95% CI 0.72–0.78) (Fig. 1A and B), where the minor allele G is more frequent in controls than in CeD cases, suggesting that the allele has a protective effect. To check for possible additional independent signals, we performed a conditional analysis on this top SNP, but did not uncover any novel significant signals (Supplementary Material, Fig. S1), indicating association to a single, common haplotype.

**Table 1.** Population-specific top SNPs compared with the meta-top SNP

Population	Cases	Controls	SNPs	BP (Hg19)	Minor allele	Major allele	MAF	MAF (cases)	MAF (controls)	OR (95% CI)	*P*	*r*^2^ rs2030519	*D*′ rs2030519
Meta-analysis	12 513	12 656	rs2030519	188119901	G	A	0.4	0.41	0.48	0.75 (0.72–0.78)	1.76E−49	1	1
UK	7728	8274	rs2030519	188119901	G	A	0.4	0.4	0.48	0.74 (0.70–0.77)	4.56E−38	1	1
Italy	1486	1270	rs9834159	188115232	T	A	0.4	0.45	0.47	0.74 (0.67–0.83)	2.11E−07	0.99	0.99
The Netherlands	1150	1173	rs60946162	188133336	T	C	0.4	0.38	0.43	0.80 (0.73–0.93)	0.002353	0.8	0.96
Spain	1131	662	rs60946162	188133336	T	C	0.4	0.44	0.49	0.78 (0.67–0.89)	0.0004836	0.8	0.96
Poland	521	541	rs2103025	188070570	T	C	0.1	0.16	0.12	1.4 (1.12–1.85)	0.004146	0.008	0.2
India	497	736	rs11923721	188100188	T	A	0.08	0.09	0.07	1.4 (1.05–2.58)	0.0207	0.045	0.91

SNP ID according to dbSNP137. MAF, minor allele frequency, based on samples from the Immunochip. The odds ratio (OR) is shown for the minor allele with a confidence interval of 95%. The *P*-value (*P*) was calculated according to a logistic regression association test. The *r*^2^ and *D*′ were calculated based on genotyped information from each population.

### Imputation confirmed the meta-analysis results

In order to test whether any non-genotyped variants were more strongly associated with CeD than the top SNP, we imputed genotypes using two different reference panels [1000 Genomes Project and Genome of the Netherlands (GoNL)]. This analysis resulted in an additional 397 SNPs (254 SNPs from the 1000 Genomes project data, 110 SNPs from the GoNL study, with 33 SNPs identified by both). We then performed a meta-analysis on both the known, as well as the imputed, SNPs. This confirmed that SNP rs2030519 was the strongest CeD-associated SNP in the *LPP* locus (*P* = 1.79 × 10^−49^, OR = 0.75, 95% CI 0.72–0.78) in this region (Fig. 1B).

### Association analysis in different populations indicated different top SNPs

Since the meta-analysis identified rs2030519 as the most strongly associated SNP, we tested whether rs2030519 was also the top SNP in the six separate populations. The top SNP was confirmed as the strongest associated SNP only in the UK population (the largest cohort) (*P* = 4.56 × 10^−38^, OR = 0.74, 95% CI 0.70–0.77), whereas in the other five populations, different top SNPs were identified (Table 1). In the Italian population, the top SNP was rs9834159 (*P* = 2.11 × 10^−7^, OR = 0.74, 95% CI 0.67–0.83), and in the Dutch and Spanish cohorts the top SNP was rs60946162 (*P* = 0.002, OR = 0.8, 95% CI 0.73–0.93 and *P* = 0.0004, OR = 0.78, 95% CI 0.67–0.89, respectively). The top SNPs in the Polish cohort (rs2103025; *P* = 0.004, OR = 1.4, 95% CI 1.12–1.85) and in the Indian cohort (rs11923721; *P* = 0.02, OR = 1.4, 95% CI 1.05–2.58) showed association in opposite directions (Table 1) to the top SNPs in the other four cohorts (UK, Italy, Netherlands and Spain). These results suggest that there could be population-specific, CeD-associated alleles.

### Haplotype analysis suggested a common CeD-associated haplotype

The existence of different CeD-associated top SNPs in different populations may suggest that these SNPs could be tagging a common functional variant on a common haplotype. We therefore included all the genotyped SNPs within a 250 kb window around the top SNP (rs2030519) from the meta-analysis, and generated haplotypes in populations that showed significant *P*-value with rs2030519 after a Bonferroni correction for the six populations (*P* < 0.008). Next, we tested the association of haplotypes with CeD using logistic regression in the separate populations. The Indian and Polish cohort were excluded from haplotype association analysis (Supplementary Material, Table S3). We therefore performed the haplotype association test in a meta-analysis of the four remaining populations together (UK, Italy, Netherlands and Spain). The biggest haplotype size (53.7 kb) was found in the Dutch cohort (Table 2) and the smallest haplotype (3.1 kb, Table 2) was found in the UK population, which was the largest cohort (Supplementary Material, Table S1). By looking at the most strongly associated haplotype in each of the European populations, we identified a core haplotype of 4.1 kb containing 14 SNPs (Table 2 and Supplementary Material, Fig. S2 and Table S4).

**Table 2.** Haplotypes analysis per population

Population	NSNP	CHR	BP1 (HG19)	BP2 (HG19)	SNP1	SNP2	HAPLOTYPE	Freq	Freq cases	Freq controls	OR	*P*	Haplotype size (kb)
The Netherlands	53	3	188083987	188137767	rs76211316	rs13319297	CTGTGCGGATATTATGGCGGCA CCCTGTACGATCTGGC**G**CAT GTTCGCCTTAA	0.38	0.3595	0.4	0.846	0.00549	53.7
Spain	43	3	188087628	188122978	rs9851967	rs2049218	TGCGGATATTATGGCGGCACCCT CGTACGATCTGGC**G**CATGTT	0.442	0.4184	0.4834	0.768	0.000168	35.3
Italy	20	3	188114458	188122978	rs12107245	rs2049218	CGTACCGATCTGGC**G**CATGT	0.453	0.4226	0.4894	0.759	5.77E−07	8.5
UK	11	3	188116784	188119901	rs9820681	rs2030519	ACGATCTGGC**G**	0.447	0.4094	0.4818	0.743	1.11E−38	3.1
Meta-analysis^a^	14	3	188116907	188121019	rs7634898	rs12634152	CCGATCTGGC**G**CAT	0.454	0.4219	0.4857	0.771	1.35E−44	4.8

NSNP is the number of SNPs per haplotype, position in base pair (BP) according with NCBI Build 37 Human Genome release 19 (HG19). SNP1 represents the SNP ID of the leftmost (5′) SNP and SNP2 is the SNP ID of rightmost (3′) SNP. In the HAPLOTYPE column, the alleles in the core haplotype are underlined. The minor allele from the top SNP rs2030519 from the meta-analysis is shown in bold. The ORs are shown for minor haplotypes. *P*-value (*P*) was calculated according to a haplotype logistic regression association test. Haplotype sizes are shown in kb.

^a^Meta-analysis without Indian and Polish cohorts.

### Seven SNPs differentiate risk and non-risk haplotypes

In order to see whether differences in the risk and protective haplotypes across populations could reduce the size of this region, we performed a haplotype logistic regression using the 14 SNPs (Table 3) in each population separately, as well as in all four populations together (Table 3). By comparing the haplotypes obtained, we found a common, associated haplotype present in all the populations (Table 3). Seven of the 14 SNPs showed minor alleles present in the protective haplotype, but absent in the risk haplotype (Table 3). This approach reduced the core haplotype down to a 2.8 kb region (chr3: 188117070–188119901, NCBI build 37) and suggests that this region might well harbor the causative variant for CeD (Fig. 1C).

**Table 3.** Haplotype association analysis in the 14 SNPs from the core haplotype

Population	rs7634898	rs7635012	rs6790260	rs28637341	rs6444284	rs6444285	rs4686483	rs4686484	rs6778720	rs2030520	**rs2030519**	rs6785284	rs1035765	rs12634152	Frequency	Freq cases	Freq controls	OR	*P*
Meta-analysis^a^	**C**	**C**	**G**	A	T	**C**	**T**	**G**	**G**	**C**	**G**	C	A	T	0.454	0.4219	0.4857	0.768	9.41E−45
	**C**	**C**	C	**T**	**C**	T	A	A	A	T	A	**T**	**T**	**C**	0.435	0.4609	0.4081	1.25	8.70E−32
	T	A	C	A	T	T	A	A	A	T	A	C	A	**C**	0.0477	0.05143	0.044	1.18	0.000115
	**C**	A	C	A	T	T	A	A	A	T	A	C	A	T	0.0619	0.06375	0.06003	1.06	0.111
UK	**C**	**C**	**G**	A	T	**C**	**T**	**G**	**G**	**C**	**G**	C	A	T	0.447	0.4094	0.4818	0.74	2.84E−38
	**C**	**C**	C	**T**	**C**	T	A	A	A	T	A	**T**	**T**	**C**	0.438	0.4673	0.41	1.28	1.45E−25
	T	A	C	A	T	T	A	A	A	T	A	C	A	**C**	0.0465	0.05092	0.0423	1.22	0.000294
	**C**	A	C	A	T	T	A	A	A	T	A	C	A	T	0.0673	0.07046	0.06442	1.09	0.051
Italy	**C**	**C**	**G**	A	T	**C**	**T**	**G**	**G**	**C**	**G**	C	A	T	0.487	0.4556	0.5248	0.755	4.12E−07
	**C**	**C**	C	**T**	**C**	T	A	A	A	T	A	**T**	**T**	**C**	0.431	0.4599	0.398	1.29	4.67E−06
	**C**	A	C	A	T	T	A	A	A	T	A	C	A	T	0.0292	0.03131	0.02677	1.2	0.262
	T	A	C	A	T	T	A	A	A	T	A	C	A	**C**	0.0483	0.05051	0.04567	1.09	0.503
The Netherlands	**C**	**C**	**G**	A	T	**C**	**T**	**G**	**G**	**C**	**G**	C	A	T	0.437	0.4143	0.4595	0.843	0.00482
	**C**	**C**	C	**T**	**C**	T	A	A	A	T	A	**T**	**T**	**C**	0.456	0.4796	0.4335	1.2	0.00277
	**C**	A	C	A	T	T	A	A	A	T	A	C	A	T	0.0575	0.05696	0.05797	0.945	0.659
	T	A	C	A	T	T	A	A	A	T	A	C	A	**C**	0.0474	0.04783	0.04646	1.02	0.879
Spain	**C**	**C**	**G**	A	T	**C**	**T**	**G**	**G**	**C**	**G**	C	A	T	0.488	0.4654	0.5257	0.783	0.000551
	**C**	**C**	C	**T**	**C**	T	A	A	A	T	A	**T**	**T**	**C**	0.4	0.4149	0.3754	1.17	0.0245
	T	A	C	A	T	T	A	A	A	T	A	C	A	**C**	0.0483	0.05275	0.04079	1.3	0.131
	**C**	A	C	A	T	T	A	A	A	T	A	C	A	T	0.0623	0.06605	0.05589	1.21	0.199

The SNP in bold is the top SNP from the meta-analysis. The minor alleles from each SNP are bold. The SNP that differentiate the risk to non-risk haplotype are highlighted in bold. OR are shown for minor haplotypes. *P*-values (P) were calculated according to a haplotype logistic regression association test.

^a^Meta-analysis without Indian and Polish cohorts.

### Annotation of established functional elements in the core haplotype region

To investigate whether the seven core-haplotype SNPs are located within known functional elements, they were subjected to HaploReg. The 2.8 kb region contained open chromatin regions, as identified in 19 cell lines of the ENCODE Project cell line panel (Fig. 1C and Supplementary Material, Table S5). Of the seven SNPs, six (rs6790260, rs6444285, rs4686483, rs4686484, rs6778720 and rs2030520) mapped to regions annotated with histone modifications H3K27ac, H3K4m1, H3k4 m2, H2A.Z and EZH2 identified in B- and T-cells (Fig. 1C and Supplementary Material, Table S5). Interestingly, SNP rs4686484 (*P* = 3.12 × 10^−49^), which is in high LD with the top-meta SNP, rs2030519 (*r*^2^ ≥ 0.9 and *D*′ = 1), maps to DNase hypersensitive sites in multiple cell lines (including B- and T-cells). In addition, it maps to overlapping DNA fragments that were individually isolated by chromatin immunoprecipitation (ChIP) using antibodies against BATF and PU.1 (Fig. 1C and Supplementary Material, Table S5). Hence, SNP rs4686484 is highly conserved (Fig. 1C, Supplementary Material, Table S5) and HaploReg suggested that this SNP changes the consensus-binding motif of the transcription factors IRF4, IRF11, Nkx2.7 and Nkx2.9 (Fig. 1D, Supplementary Material, Table S5).

In order to test whether the SNP rs4686484 can be prioritized as the only candidate SNP without performing haplotype analysis, we subjected 174 SNPs present in the original 70 kb region to HaploReg annotation (Supplementary Material, Fig. S4). We found 12 SNPs in high LD with rs4686484 (*r*^2^ ≥ 0.7 and *D*′ = 1) overlapping with regulatory elements characterized by open chromatin, histone modification and transcription factor binding. However, it is not clear which of these 12 SNPs are functionally involved in CeD pathogenesis. Therefore, the fine mapping by haplotype analysis helped us to prioritize the most likely functional SNP even though the associated region contained more than one SNP with similar regulatory potential.

### Sanger sequencing to identify novel variants

In order to test whether the highly conserved regulatory region contained novel variants in LD with SNP rs4686484, we sequenced 391 bp around this functional SNP (chr3: 188118290–188118680, NCBI build 37) in 210 Dutch individuals (117 cases and 93 controls). This did not identify any novel variants in the region, which was in concordance with GoNL data (11). Although two SNPs (rs147428170 and rs148483836) are located in this region (according to the 1000 Genomes Project), we were not able to confirm them in the 210 individuals we sequenced, probably because these two SNPs are very rare (minor allele frequency, MAF < 0.001) in the CEU population. We therefore consider SNP rs4686484 to be the main functional candidate (Supplementary Material, Table S6).

### Low expression of the *LPP* gene in biopsies from CeD patients

In order to test whether *LPP* gene levels are affected in the CeD status, microarray expression analysis was performed on 25 duodenal biopsies (12 celiac patients with villous atrophy and 13 healthy controls) (Supplementary Material, Methods). This showed a lower expression of the *LPP* gene in biopsies of celiac patients with villous atrophy (Marsh III stage) compared with healthy controls (Supplementary Material, Fig. S3). These results suggest *LPP* is a causative gene in the pathogenesis of CeD.

## DISCUSSION

We aimed to refine a 70 kb LD region of the *LPP* gene, previously reported as the strongest non-HLA-associated locus in CeD, and to identify possible functional variants. We analyzed genotype data obtained with Immunochip in six-independent populations in this region (6). A meta-analysis on the six sets of genotyped and imputed data confirmed that the strongest association was to the SNP rs2030519 (*P* = 1.79 × 10^−49^) located in intron 2 of the *LPP* locus (Fig. 1A and B). Subsequent conditional analysis on this SNP indicated no other independent associations and suggested the presence of a single, common risk haplotype in this locus (Supplementary Material, Fig. S1).

It has been suggested that disease etiology is common between populations, but that risk variants are often population specific (12). Here, we performed a population-specific association analysis to test the genetic heterogeneity between six populations. Except for the UK population, their top SNPs differed from the meta-analysis top SNP (Table 1). In addition, except for the Polish top SNP, the top SNPs of the other populations were in LD with the meta-analysis top SNP (Table 1), suggesting a common-associated haplotype in these populations.

Haplotype analysis using different populations has been shown to be a suitable strategy for fine-mapping associated regions (13). Since haplotypes characterize the exact organization of alleles along the chromosome (14), more information can be incorporated into the association tests by constructing haplotype blocks from SNPs (15). Accordingly, in this study, we were able to find a core haplotype of 4.1 kb by comparing the most strongly-associated haplotype per population in four Caucasian cohorts, which indicated a common associated haplotype between these populations (Fig. 1C, Table 2). In addition, we observed that the sample size of the study cohort is very important to narrow down the associated regions. By analyzing only the two largest populations (UK and Italy), we found a haplotype with a more similar size of 4.8 kb than when we investigated all the populations together (Supplementary Material, Fig S2). However, only by adding the other populations, we were able to compare risk and protective haplotypes, and finally pinpoint seven SNPs that differentiate the risk haplotype from the non-risk haplotype across different populations (Fig. 1C, Table 3). This helped us to refine the associated region down to 2.8 kb, where a functional variant might be located.

It has been shown that there is an enrichment of regulatory elements from the ENCODE project overlapping SNPs identified by GWAS (10,16). To prioritize SNPs in the 2.8 kb region that could have a functional effect, we intersected all seven SNPs in the 2.8 kb region with regulatory information available from the ENCODE Project (10). In the end, we were able to identify six out of seven SNPs that mapped to a regulatory region, either to DNase hypersensitive sites or regions with enhancers histone modifications (Fig. 1C, Supplementary Material, Table S4). One SNP, rs4686484, directly overlapped these two regulatory sites and also mapped to transcription-factor-binding sites (Fig. 1C). The G allele at rs4686484 was predicted to interfere with the consensus motives of transcription factors IRF4, IRF11, Nfx2.7 and Nfx2.9 (Fig. 1D). Interestingly, IRF4 has been described as interacting with PU.1 (17), and since the G allele of the functional SNP interferes with IRF4 binding, it is possible that this SNP could affect IRF4-mediated PU.1 binding. Whether this alteration would increase or decrease the expression of *LPP* remains to be tested. Moreover, the *IRF4* gene was also reported to be associated with CeD (6), and it could be that it somehow interacts with the *LPP* gene. To investigate this, we used Genenetwork (www.genenetwork.nl) and DAVID (18); however, we did not find any shared pathways between *LPP* and *IRF4* (results not shown).

The lack of any novel variants on sequencing, as well as the many overlapping layers of regulatory information within this highly conserved regulatory region, confirmed rs4686484 as the most likely functional SNP (Supplementary Material, Table S6). However, we did not find any eQTL effect for SNP rs4686484 in a peripheral blood dataset of 1240 samples (19). We were therefore unable to establish a causal link between the prioritized functional SNP and the *LPP* gene. Nonetheless, microarray analysis in duodenal biopsies showed that the *LPP* gene is down-regulated in severe CeD cases (villous atrophy, Marsh III) compared with healthy individuals (Supplementary Material, Fig. S3). We examined the expression level of *LPP* in biopsies from individuals genotyped for the rs4686484 SNP, but found no significant difference, most likely due to our very limited sample size (*n* = 25) (results not shown). Nevertheless, this result is in agreement with a previous study that also showed lower expression of the *LPP* gene in biopsies of CeD patients (20), suggesting that *LPP* is the most plausible causative gene in this locus.

The fact that many regulatory elements in B-cells from the ENCODE project overlap with the fine-mapped region (Fig. 1C, Supplementary Material, Table S5) suggests that our functional SNP may impact gene expression in B-cells. Although CeD is known to be mainly a T-cell disorder, the role of B-cells in the pathogenesis of CeD is being increasingly recognized (21,22). It has been suggested that B-cells, in combination with the enzyme transglutaminase 2, can present gluten peptides to gluten reactive T-cells (22). Our data suggest that B-cells are an important cell type for performing functional assays to better understand the role of the *LPP* locus in CeD pathogenesis.

Since the prioritized SNP rs4686484 has a regulatory function, it may affect other genes outside of the LD block as well. In this context, the *BCL6* gene, located 658.7 kb away from the candidate SNP, is a plausible candidate. Recently, a meta-analysis for allergic sensitization identified an SNP (rs9865818) located in the *LPP* gene, and in LD with our candidate SNP rs4686484 (*r*^2^ = 0.75, *D*′ = 0.93), that showed a *cis*-eQTL effect specifically for B-cells in the *BCL6* gene (23). This suggests that it is still possible that the SNP rs4686484, or an SNP in the same haplotype, can act by regulating *BCL6* specifically in B-cells. Additionally, to support this idea, it is known that *IRF4* and *BCL6* have a very close interaction. We therefore analyzed the expression data of *BCL6* in biopsies similar to *LPP*, but found no difference between CeD and healthy control biopsies (Supplementary Material, Fig S5). This finding suggests that *LPP* is a candidate gene. However, we do not have any conclusive evidence that links our possible causative SNP with the *LPP* gene. Hence, more eQTL studies in B-cell-specific datasets are necessary to establish a true causal gene.

In summary, we were able to narrow down an intronic region strongly associated with CeD from 70 to 2.8 kb. By integrating data from the ENCODE project with our findings, we identified six SNPs that overlap regulatory sites (Fig. 1C), with one, rs4686484, having a possible biological function. In addition, gene expression data from patient biopsies suggested that the *LPP* gene might be involved in the pathogenesis of CeD. Further functional studies are therefore warranted to validate our findings.

## MATERIALS AND METHODS

### Study population

The CeD study population (12 513 patients and 12 656 controls) included the samples described previously (6), with additional samples in each population that had since become available (Supplementary Material, Table S1). Affected individuals were diagnosed as patients with CeD according to standard clinical, serological and histo-pathological criteria, including a small intestinal biopsy (24). DNA samples were isolated from blood, lympho-blastoid cell lines or saliva, as indicated by Trynka *et al*. (6). The details of the SNP probes present on the Immunochip array, and of the genotyping and quality control filters, have been reported previously (6). All the subjects sampled for this project provided informed consent, and the study was approved by the ethics committees or institutional review boards of the hospitals where the samples were collected.

### Association analyses

A meta-analysis was performed in the 70 kb *LPP* LD region (chr3: 188069360–188139629, NCBI build 37) using PLINK (25). Gender and the country of origin were included as covariates. PLINK was also used for a stepwise, conditional logistic regression of the most associated SNPs showing the highest meta-analysis association *P*-value. In order to assess the association per population, we performed a logistic regression on each population separately, using gender as a covariate.

### Imputation

SNPs were imputed across the *LPP* LD region using data from the 1000 Genomes Project (26) and from the GoNL (11) as reference panels. Before imputation, the data were filtered for SNPs with an MAF >0.01 and deviations from Hardy–Weinberg equilibrium in controls (*P* > 0.0001), and subsequently aligned to the forward strand. The data were analyzed in batches comprising 500 samples and 2000 SNPs, with an overlap of 500 SNPs. Phasing was performed using MACH 1.0.18 (27) applying 20 rounds of 200 states. We used MOLGENIS compute (28) imputation pipeline to generate our scripts and monitor the imputation. IMPUTE2 (29) was used in both reference panels, applying its default options. Finally, a meta-analysis on all the cohorts together was performed, as described above.

### Haplotype analysis

Haplotype analyses were performed using PLINK v1.7 (25), where haplotype blocks were generated within a 250 kb window harboring the most significant SNP from the meta-analysis. Next, a Bonferroni correction was performed for the six populations tested and only the populations that had the top meta-analysis SNP with significant *P*-value (0.008) were included for further analysis. Afterwards, a haplotype logistic regression analysis in each population was performed. In order to see differences in the risk versus non-risk haplotypes, SNPs located in the core haplotype (an overlapping, shared haplotype region in all populations) were used to perform a haplotype association test per population.

### Functional annotation of variants

The regulatory regions overlapping with the identified SNPs and proxies (*r*^2^ ≥ 0.9, 1000 Genomes project-CEU), and present in the fine-mapped haplotype, were annotated using HaploRegv2 (30) default settings. HaploReg takes the SNPs on the haplotype block, and using LD information from the 1000 Genomes Project, intersects these SNPs with functional regions identified by the ENCODE project (10). In addition, it predicts the effects of an SNP in a regulatory region based on a library of position-weighted matrices collected from JASPER (31), TRANSFAC (32) and protein-binding microarray experiments (33–35). HaploReg v2 also provides annotation of mammalian conservation based on GERP (36) and Si-phy (37) algorithms. UCSC Genome Browser was used to look for intersections between SNPs and regulatory elements from the most recent data of the ENCODE project (10).

## SUPPLEMENTARY MATERIAL

Supplementary Material is available at *HMG* online.

## FUNDING

This work was supported by funding from the European Research Council under the European Union's Seventh Framework Programme (FP/2007-2013)/ERC Grant Agreement (n. 2012-322698) to C.W., the Dutch Digestive Disease Research Foundation (MLDS, WO11-30 to C.W.), the Netherlands Organization for Scientific Research (NWO-VICI grant 918.66.620) to C.W. and the Capes Foundation, Ministry of Education of Brazil, for a fellowship (Proc. no. BEX 0891-10-0) to R.A. This study makes use of data generated by the Genome of the Netherlands Project, which is funded by the Netherlands Organization for Scientific Research (award number 184021007, 9 July 2009) and made available as a Rainbow Project of the Biobanking and Biomolecular Research Infrastructure Netherlands (BBMRI-NL). Samples where contributed by Lifelines (http://lifelines.nl/lifelines-research/general), the Leiden Longevity Study (www.healthy-ageing.nl; www.langleven.net), the Netherlands Twin Registry (NTR, www.tweelingenregister.org), the Rotterdam studies (www.erasmus-epidemiology.nl/rotterdamstudy) and the Genetic Research in Isolated Populations program (www.epib.nl/research/geneticepi/research.html#gip). The sequencing was carried out in collaboration with the Beijing Institute for Genomics (BGI). Funding to pay the Open Access publication charges for this article was provided by the European Research Council under the European Union's Seventh Framework Programme (FP/2007-2013)/ERC Grant Agreement (n. 2012-322698).

## Supplementary Material

Supplementary Data
