# Supplementary material for: Fine mapping of the celiac disease-associated LPP locus reveals a potential functional variant
Source: Hum Mol Genet. 2013 Dec 11;23(9):2481–9. doi: 10.1093/hmg/ddt619 (PMC3976328; doi:10.1093/hmg/ddt619)
Supplement: Supplementary Data [file supp_23_9_2481__index.html]

Fine mapping of the celiac disease-associated LPP locus reveals a potential functional variant — Fine mapping of the celiac disease-associated LPP locus reveals a potential functional variant — Supplementary Data 

# Fine mapping of the celiac disease-associated *LPP* locus reveals a potential functional variant

## Supplementary Data

Supplementary Data

**Files in this Data Supplement:**

- Supplementary Figures - docx file
- Supplementary Tables - xlsx file
