# Supplementary material for: Fine mapping of the celiac disease-associated LPP locus reveals a potential functional variant
Source: Hum Mol Genet. 2013 Dec 11;23(9):2481–9. doi: 10.1093/hmg/ddt619 (PMC3976328; doi:10.1093/hmg/ddt619)
Supplement: Supplementary Data [file supp_ddt619_ddt619supp_figs.docx]

**SUPPLEMENTARY MATERIAL**

**Fine-mapping of the celiac disease-associated *LPP* locus reveals a potential functional variant**

**SUPPLEMENTARY METHODS**

**Sequencing of the regulatory region**

Sanger sequencing was performed in 210 Dutch individuals (117 cases 93 controls). Specific PCR primers (available upon request) were designed to cover the target region of 391 bp (chr3: 188,118,264-188118779 NCBI Build 37) around the functional candidate SNP rs4686484. PCR amplifications were performed in 96-well plates in a volume of 7 uL. DNA sequencing analysis was performed using the software Mutation Surveyor (1).

**Analysis of biopsies**

Biopsy sampling, RNA isolation, and details of microarray hybridisation have been previously described (2). Intestinal biopsies from 12 celiac disease patients with histological classification of Marsh III (MIII), and duodenum biopsies from 13 healthy individuals were investigated according to UEGW criteria (3). Total RNA was isolated from these biopsies using TRIzol (Gibco/Life Technologies, Rockville, Maryland, USA) following the manufacturer’s protocol. The normalized expression values were stratified according to the phenotypes (CeD versus controls) and the significant difference was tested using Wilcoxon rank test (implemented in R).

**REFERENCES:**

1. Minton JA, Flanagan SE, Ellard S. (2011) Mutation surveyor: software for DNA sequence analysis. Methods Mol. Biol., **688**:143-153.

2. Diosdado B, Wapenaar MC, Franke L, Duran KJ, Goerres MJ, Hadithi M, Crusius JB, Meijer JW, Duggan DJ, Mulder CJ, Holstege FC, Wijmenga C. (2004) A microarray screen for novel candidate genes in coeliac disease pathogenesis. Gut, **53**:944-951.

3. Mulder C, Rostami K, Marsh MN. (1998) When is a coeliac a coeliac? Gut, **42**:594.

4. Trynka, G., Hunt, K.A., Bockett, N.A., Romanos, J., Mistry, V., Szperl, A., Bakker, S.F., Bardella, M.T., Bhaw-Rosun, L., Castillejo, G. *et al.* (2011) Dense genotyping identifies and localizes multiple common and rare variant association signals in celiac disease. *Nat. Genet.*, **43**:1193-11201.

5. Husby, S., Koletzko, S., Korponay-Szabo, I.R., Mearin, M.L., Phillips, A., Shamir, R., Troncone, R., Giersiepen, K., Branski, D., Catassi, C. *et al.*(2012) European Society for Pediatric Gastroenterology, Hepatology, and Nutrition guidelines for the diagnosis of coeliac disease. *J. Pediatr. Gastroenterol. Nutr.,* **54**:136-160.

**SUPPLEMENTARY FIGURES**


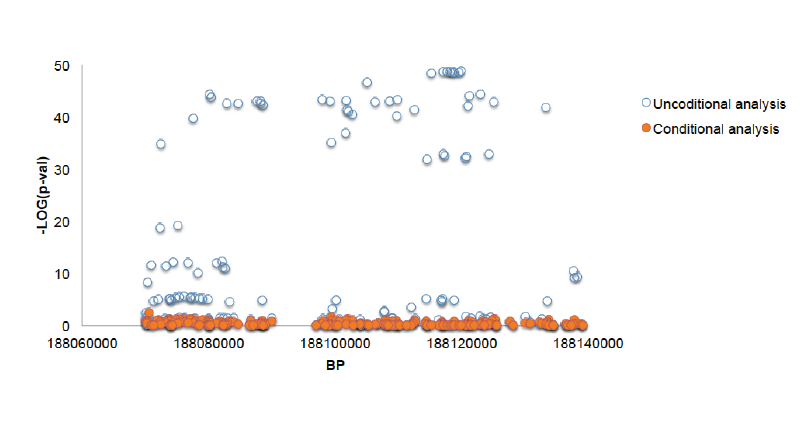


**Figure S1**: Conditional analysis and unconditional analysis on the top meta-analysis SNP rs2030519. Open blue circles represent SNPs identified by unconditional meta-analysis. Orange dots are the SNPs after conditional analysis on the top meta SNP.


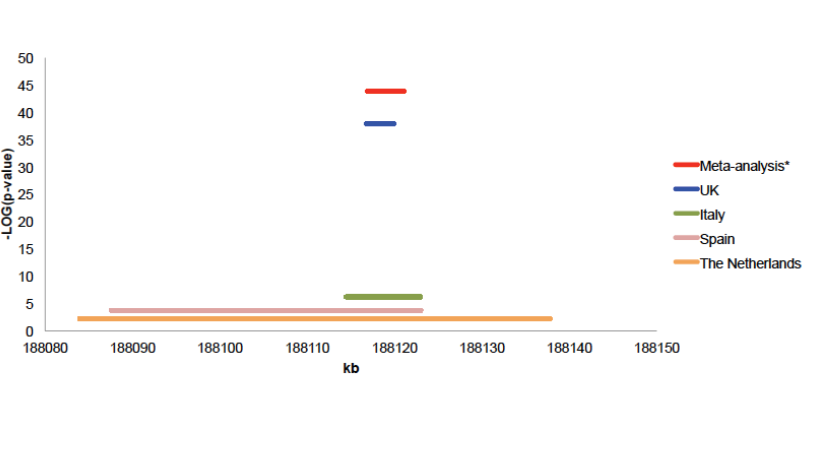


**Figure S2**: Haplotype association per population and meta-analysis. Every line represents the stronger associated haplotype per population, as well as, in the meta-analysis. The red line on top is the stronger haplotype in the meta-analysis without the Indian population. The orange line on the bottom represents the most associated haplotype in the Dutch population.

*Meta-analysis without the Indian and Polish populations.


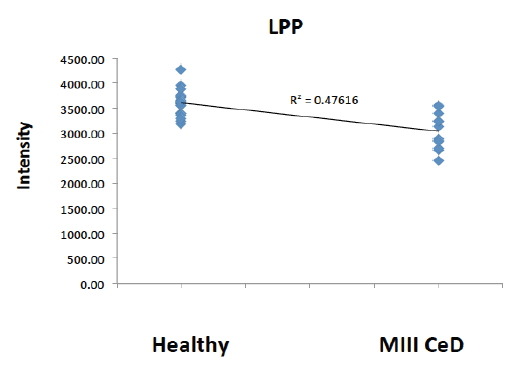


**Figure S3:** Microarray analysis in biopsies of celiac patients and healthy individuals. Blue dots represent the biopsies from each individual.

MIII: Marsh III, CeD: celiac disease.


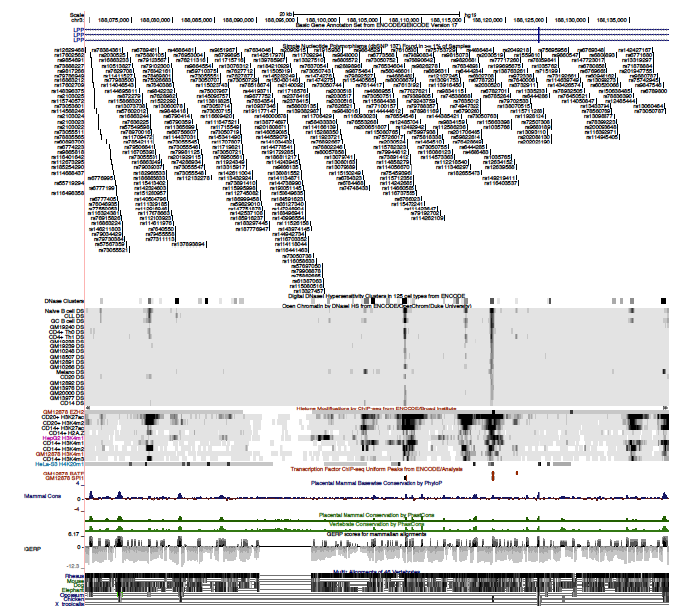


**Figure S4:** UCSC genome browser. SNPs in the 70kb original region intersected with the ENCODE data and evolutionary conserved sites. Signal peaks are depicted as darker regions.


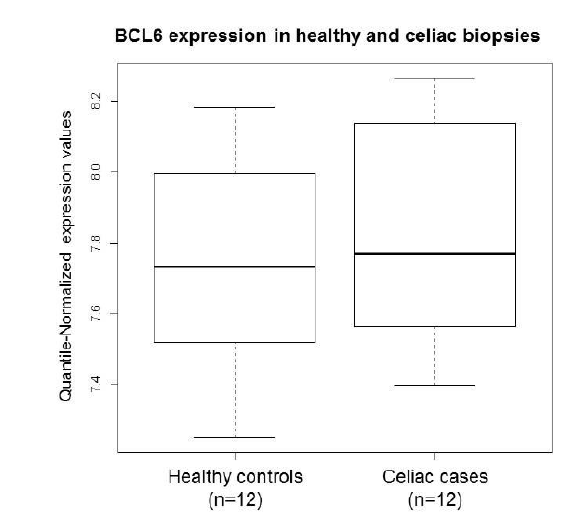


**Figure S5:** Microarray analysis in biopsies of celiac patients and healthy individuals. Box plots shows quartile normalized expression values for *BCL6* in healthy and celiac patients.
